# Chemoprevention in oral leukoplakia: challenges and current landscape

**DOI:** 10.3389/froh.2023.1191347

**Published:** 2023-05-24

**Authors:** Victor de Mello Palma, Natalia Koerich Laureano, Luiza Abrahão Frank, Pantelis Varvaki Rados, Fernanda Visioli

**Affiliations:** ^1^ Oral Medicine Department, School of Dentistry, Universidade Federal do Rio Grande do Sul (UFRGS), Porto Alegre, Brazil; ^2^ Programa de Pós-Graduação em Ciências Farmacêuticas, Universidade Federal do Rio Grande do Sul (UFRGS), Porto Alegre, Brazil; ^3^ Experimental Center Research, Hospital de Clínicas de Porto Alegre, Porto Alegre, Brazil

**Keywords:** chemoprevention, oral cancer, potentially malignant disorders, leukoplakia, secondary prevention

## Abstract

Oral potentially malignant disorders have the potential to transform into oral cancer. Oral leukoplakia is a prevalent OPMD with a 9.8% malignant transformation rate. The standard management for OL involves surgical excision, but its efficacy in preventing clinical recurrence and malignant transformation is limited. Therefore, alternative strategies such as chemoprevention modalities have emerged as a promising approach to inhibit the carcinogenesis process. The aim of this review was to identify human studies that investigated the effectiveness of chemopreventive agents in preventing the progression of oral leukoplakia and to provide guidance for future research. Several systemic and topical agents have been evaluated for their potential chemopreventive effects in oral leukoplakia. Systemic agents that have been investigated include vitamin A, lycopene, celecoxib, green tea extract, ZengShengPing, Bowman Birk inhibitor, beta-carotene, curcumin, erlotinib, and metformin. In addition, topical agents tested include bleomycin, isotretinoin, ONYX-015 mouthwash, ketorolac, and dried black raspberry. Despite numerous agents that have already been tested, evidence supporting their effectiveness is limited. To improve the search for an ideal chemopreventive agent for oral leukoplakia, we propose several strategies that can be implemented. Oral leukoplakia chemoprevention presents a promising opportunity for decreasing the incidence of oral cancer. Identifying new chemopreventive agents and biomarkers for predicting treatment response should be a focus of future research.

## 1. Introduction

Oral potentially malignant disorders (OPMD) are characterized by tissue changes that are more likely to transform into oral cancer than normal tissues (1). Oral cancer refers to a group of malignant neoplasms that affect the oral mucosa, and it represents a significant public health concern worldwide (2). The most common type of oral cancer is the oral squamous cell carcinoma (OSCC), which accounts for over 90% of malignant tumors in the oral cavity (3).

Although OPMD are not always precursors of oral cancer, it is estimated that 7% of SCCs are preceded by this type of lesions (4). However, this data might have been underestimated due to underdiagnosis of OPMDs. Oral leukoplakia (OL) is one of the most prevalent OPMD (5), for which there is currently no effective therapeutic approach to prevent its progression to cancer. OL is defined as “A predominantly white plaque of questionable risk having excluded (other) known diseases or disorders that carry no increased risk for cancer” (6).

The malignant transformation (MT) rate is 9.8%, with a mean transformation length of 3.2 years (range: 1.8–5.1 years) (5). When presenting epithelial dysplasia, this rate increases to 15.3% (7). The OL patient's profile with higher risk for MT is: female gender, non-smokers, long duration of leukoplakia, location on the tongue and/or floor of the mouth, size >200 mm^2^, non-homogeneous type, presence of epithelial dysplasia, and DNA aneuploidy (8).

The standard management of leukoplakia is surgical excision, and follow-up is guided by histological diagnosis: 6-month intervals for OL without epithelial dysplasia and 3-month intervals for OL with epithelial dysplasia. However, this approach has shown limited efficacy in preventing clinical recurrence and MT. The question that remains is whether this follow-up should be lifelong (9).

Other therapeutic modalities, such as cryosurgery, laser surgery (10), topical or systemic retinoids (11, 12), adenovirus-containing mouthwash (13), and photodynamic therapy (14), have been tested. While there is no clear scientific evidence supporting the effectiveness of these modalities in preventing OL MT (15), healthcare professionals consider treating leukoplakia a safe practice (9, 16) regardless of histological changes, as it is an attempt to prevent MT.

Different chemopreventive agents have been tested in several studies as an alternative treatment to prevent leukoplakia MT. However, the efficacy of these agents remains limited as all chemoprevention trials conducted so far have shown limited success (17, 18). Despite over three decades of research on chemoprevention of oral cancer, a pharmacological strategy that can be deemed as a standard of care has not yet been established (19).

This review aims to identify human studies that examined the effectiveness of chemopreventive agents in preventing OL progression. By synthesizing the available evidence, an overview of the current state of OL chemoprevention is provided, which can help identify gaps in the literature and guide future research to develop effective and safe chemopreventive strategies for oral cancer.

## 2. Concepts in chemoprevention

The emergence of chemoprevention for OSCC was based on the failure of conventional therapies for definitive control of OPMD. Chemoprevention denotes the application of either natural or synthetic chemical agents, applied topically or systemically, which are used to preclude the advancement or genesis of oral cancer (20, 21).

Chemoprevention is a promising method to reduce oral cancer incidence and mortality rates, especially in high-risk populations. Chemoprevention can restrain the development of cancer by acting on various stages of carcinogenesis. At the initiation, it can block DNA damage, whereas at the tumor promotion and progression stages, it can reverse or suppress the proliferation of premalignant cells (22, 23). Thus, chemoprevention can be formulated to either inhibit, halt, or reverse these processes (24).

From a conceptual perspective, chemoprevention can be categorized as primary, secondary, and tertiary. Primary chemoprevention focuses on healthy individuals with a high probability of developing cancer. Secondary chemoprevention targets individuals with established premalignancy, such as OPMD. Tertiary chemoprevention aims to prevent recurrence or the emergence of secondary tumors in individuals who have been previously diagnosed and treated cancer (25). This review will focus on the role of secondary chemoprevention in preventing OL progression, relapse, and its MT.

A search was conducted in the PubMed database using the strategy “oral leukoplakia AND chemoprevention”, with filters applied for Clinical Trial and Randomized Controlled Trial. A total of 37 articles were retrieved. Inclusion criteria were limited to human studies and articles published in English. Since this is a mini review, only the most relevant studies of each chemoprevention agent were selected (recency, higher sample size, follow-up period). However, a manual search of the references cited in these articles was conducted to identify relevant older and classic studies to provide a more comprehensive overview of the topic. Finally, a total of 19 articles were included in this mini review.

## 3. Current landscape in chemoprevention of oral cancer

### 3.1. Systemic chemoprevention

Systemic chemoprevention refers to the use of agents that are given orally or intravenously (22). The goal is to reduce the risk of oral cancer by targeting molecular pathways involved in carcinogenesis, including those regulating cell proliferation, cell cycle, apoptosis, angiogenesis, and inflammation (26).

The main studies in this subject are available at Table 1. Among the systemic chemopreventive agents used are vitamin A (27), lycopene (28), celecoxib (29), green tea extract (30), zengshengping (31), bowman-birk (32), beta-carotene (33), curcumin (34), erlotinib (35), and metformin (36). All of these studies (100%) were randomized controlled trials (RCTs). The sample sizes ranged from 23 to 223 participants. The primary clinical response, which aimed to reduce or eliminate the lesions, was the most frequently assessed outcome, reported in 90% of the trials, although only four studies observed a statistically significant benefit for this outcome (27, 28, 31, 34). Histological analysis was the second most reported outcome, mentioned in 60% of the studies, however most of the studies did not show improvement in this outcome. In addition, 40% evaluated protein or molecular biomarkers. Adverse effects were reported in 60% of the studies. The follow-up time varied from 12 weeks to 5 years.

**Table 1:** Studies that employed systemic chemoprevention agents to treat oral leukoplakia.

Reference/design study	Chemopreventive protocol/sample (*n*)	Outcomes/biomarkers	Main results	Side effects	Follow-up
Stich et al., (27) RCT	Vitamin A capsules (200.000 IU/week) (*n* = 21) Placebo (*n* = 33) 2x week for 6 months	Clinical and histological	CR: Vitamin A 57.1% Placebo 3% (*p* < 0.001) HR: 85% showed thinning of the spinous layer	None	6 months
Singh et al., (28) RCT	A-Lycopene 8 mg/day (*n* = 20) B-Lycopene 4 mg/day (*n* = 20) C-placebo (*n* = 18) 1x day for 3 months	Clinical and histological	CR: (A) 80% (*p* < 0.001) (B) 66.25% (*p* < 0.001) (C) 12.5% HR: (A-B) (*p* < 0.05) (B-C) (*p* < 0.05) (A-C) (*p* < 0.001)	None	2 months
Papaclimitrako-poulou et al., (29) RCT	Celecoxib 100 mg (*n* = 17) Celecoxib 200 mg (*n* = 15) Placebo (*n* = 18) 2x day for 12 weeks	Clinical, histological Protein biomarker: COX-2	CR: placebo 33.3% Celecoxib 100 mg 41.2% Celecoxib 200 mg 20% (*p* = 1.0) HR: placebo 20% Celecoxib 100/200 mg 28% (*p* = 0.71) COX-2 (p = 0.046)	Dizziness, diarrhea, abdominal pain, oral pain, flatulence, nausea, headache, sinusitis, and sore throat	12 weeks
Tsão et al., (30) RCT	Green tea extract (GTE) 500 mg (*n* = 11) 750 mg (*n* = 9) 1 g (*n* = 10) Placebo (*n* = 11) 3x day for 12 weeks	Clinical, histological, Protein biomarkers: VEGF, p53, p16, Ki67 and CD1	CR: placebo 18.2% GTE 50% (*p* = 0.09) HR: placebo 9.1% GTE 21.4% (*p* = 0.68) VEGF (*p* = 0.04) p53 (*p* = 0.004)	Insomnia, headache, nausea, and nervousness	27.5 months
Sun et al., (31) RCT	ZengShengPing (ZSP) 4 tables 0.3 g (*n* = 59) Placebo (*n* = 53) 3x day for 8–12 months	Clinical, Protein biomarkers: PCNA and Agnor	CR: placebo 17% ZSP 67.8% (*p* < 0.01) PCNA-labeling index (*p* < 0.05) Agnor (*p* < 0.05)	Not reported	3 months
Armstrong et al., (32) RCT	Bowman-birk (BBIC) 30° C.I. units (*n* = 67) Placebo (*n* = 65) 2x day for 6 months	Clinical and histological Protein biomarker: neu	CR: placebo 30% BBIC 28% (*p* < 0.81) HR: no difference (*p* > 0.88)	None	6 months
Nagão et al., (33) RCT	Beta-carotene (B) 10 mg + Vitamin C (VC) 500 mg (*n* = 23) Control Vitamin C 500 mg (*n* = 23) 1x day for 1 year	Clinical and risk for malignant transformation	CR: control 4.3% B + VC 17.4% (*p* = 0.346) RMT: 0.77 (*p* = 0.580)	None	60 months
Kuriakose et al., (34) RCT	Curcumin 3.6 g (*n* = 111) Placebo (*n* = 112) 1x day for 6 months	Clinical and histological	CR: placebo 55.3% Curcumin 67.5% (*p* = 0.03) HR: placebo 20.7% Curcumin 22.5% (*p* = 0.71)	Anemia, skin/subcutaneous tissue disorders and hypertension	12 months
William et al., (35) RCT	Erlotinib 150 mg (*n* = 75) Placebo (*n* = 75) 1x day for 12 months	Oral cancer free survival (CFS), Protein biomarker: EGFR, Molecular biomarker: LOH	CFS: placebo 74% Erlotinib 70% (*p* = 0.45) CFS/LOH (+) 74% CFS/LOH (−) 87% (*p* = 0.01)	Diarrhea, fatigue and mucositis	35 months
Gutkind et al., (36) CT	Single arm: Metformin (500 mg for 1 week, 1.000 mg for 1 week and 2.000 mg for 10 weeks) 1x day	Clinical, histological, Protein biomarkers: mTOR, Ki67, p53, p16, ps6, PTEN, Molecular biomarkers: *EGFR*, *OCT3*, *TP53*, *HRAS, NOTCH1, CDKN2A, PIK3CA, CASP8*	CR: 17% HR: 60%	Gastrointestinal pain and discomfort, abdominal pain, bloating, dyspepsia, pain, and stomach pain	3 months

Considering clinical and histological outcomes, only one systemic chemopreventive agent study demonstrated superiority over placebo. Singh et al. (28) conducted an RCT investigating the effects of lycopene on OL, with participants receiving either 8 mg or 4 mg capsules for 3 months (28). A dose-dependent clinical and histological improvement was detected. Lycopene appears to be a promising antioxidant for treating OL by protecting cells against damage and preventing the progression of dysplasia. However, a short two-month follow-up was reported, making it unclear if a long-term effect can be achieved.

Taking into consideration only the clinical outcome, some chemopreventive agents have shown promise as they were superior to placebo: vitamin A resulted in a complete remission rate of 57.1% of OL without side effects (27); ZengShengPing (ZSP) extract showed a positive clinical response in 67.8% of patients, with lesion size decreasing by over 50% after three months of treatment (31); and curcumin improved clinical outcomes in 67.5% of lesions in a RCT, however, side effects such as anemia, skin/subcutaneous tissue disorders, and hypertension were reported (34).

The combination of beta-carotene and vitamin C did not show significant clinical superiority when compared to placebo. Notably, this study also assessed the risk of MT of OL over a 5-year follow-up period (33). The Bowman Birk Inhibitor concentrate (BBIC), also did not result in any significant difference between the placebo and treatment arms regarding clinical response and histological grade or the levels of neu protein, which were assessed as a treatment response biomarker (32).

In recent years, there has been a growing interest in using biomarkers as a tool to improve the efficacy of response detection. These markers can provide valuable information about the biological processes underlying the development of cancer, allowing researchers to identify higher-risk individuals and monitor the response to chemopreventive interventions (37).

First, protein biomarkers were included as secondary outcomes, to investigate both the treatment's efficacy as well as their potential to predict the evolution of the OL. In a randomized phase II pilot study, Papadimitrakopoulou et al. (29) demonstrated that doses of COX-2 inhibitor (celecoxib 100 and 200 mg twice daily) were ineffective regarding clinical and histological outcomes. However, COX-2 expression was found to correlate with OL progression (29). Tsao et al. (30) investigated the expression of VEGF, p53, p16, Ki67, and CD1 after administering green tea extract (GTE). The clinical and histological response rate was higher after GTE in a dose-response effect, but without statistical significance and with insomnia/nervousness reported as side effects (30).

More recently, molecular biomarkers were also incorporated. William et al. (35) in a RCT testing erlotinib, an EGFR tyrosine kinase inhibitor, validated loss of heterozygosity (LOH) as a risk marker for OL MT and its association with an increased number of EGFR (epidermal growth factor receptor) copies. However, erlotinib did not improve cancer-free survival in high-risk patients with LOH-positive OL or with a high number of EGFR copies (35). Gutkind et al. (36) conducted a phase IIa clinical trial in individuals with OL and oral erythroplakia to evaluate metformin's potential to target PI3K/mTOR signaling for MT prevention. The histological response, as well as saliva and blood sample collection, protein expression, and exome sequencing were assessed for outcome analysis. After 12 weeks of metformin treatment, 17% of clinical and 60% of histologic response were observed. Pathogenic mutations, compatible with those found in HPV negative HNSCC, were detected. However, none of the mutated genes were significantly associated with the metformin treatment response (36).

### 3.2. Topical chemoprevention

Site-directed chemoprevention is an alternative for managing OL since it is a low-risk and non-invasive strategy. Topical therapies have advantages, such as reducing the toxicity of chemical agents, and can be applied beyond the clinical margins of the lesions to decrease or even eliminate the risk of relapses (38).

Among the topical chemopreventive agents used are bleomycin (39, 40), isotretinoin (41–43), ONYX-015 (adenovirus) (13), ketorolac (cyclooxygenase inhibitor) (44), and dried black raspberries (45) (Table 2). Of the studies included in this review that used topical chemopreventive agents for OL, only 38% were RCTs. The sample sizes ranged from 10 to 57 participants. The primary clinical response was the most frequently assessed outcome (88%), although only two studies observed statistical significant benefit for this outcome (40, 45), followed by histological responses (63%), where only one study showed statistical difference (45). Additionally, 50% of the studies included the evaluation of protein or molecular biomarkers. Adverse effects were reported in 62% of the studies. The follow-up time varied from 3 months to 10 years.

**Table 2:** Studies that employed topical chemoprevention agents to treat oral leukoplakia.

Reference/design study	Chemopreventive protocol/sample (*n*)	Outcomes/biomarkers	Main results	Side effects	Follow-up
Wong et al., (39) CT	Bleomycin 0.5% (*n* = 5) Bleomycin 1% (*n* = 7) 1x day for 2 weeks	Clinical and histological	CR: 1% - 3 cases complete 0.5% - 2 cases partial HR: 0.5% - no improvement 1% - 3 cases complete	Burning, irritation, and erosion	23 months
Epstein et al., (40) RCT	Bleomycin 1% (*n* = 10) Placebo (*n* = 12) 1x day for 2 weeks	Clinical and histological	CR: Bleomycin 50% Placebo 8.3% (*p* = 0.001) HR: Bleomycin 60% Placebo 16.6% (*p* = 0.094)	Burning, pain, erythema with erosion	22 months
Piattelli et al., (41) CT	Isotretinoin 0,1% gel (*n* = 6) Placebo (*n* = 3) 3x day for 4 months	Clinical Protein biomarker: bcl-2 and Apoptotic analysis	CR: 10% bcl-2 (*p* = 0.134) Apoptotic bodies (*p* = 0.0193)	None	4 months
Tetê et al., (42) CT	Isotretinoin 0,1% gel Placebo (*n* = 15) 3x day for 4 months	Clinical Protein biomarker: bcl-2 and Apoptotic analysis	CR: 3 cases bcl-2 (*p* = 0.132) Apoptotic bodies (*p* = 0.0193)	Not reported	Not reported
Rudin et al., (13) CS	Mouthwash ONYX-015 for p53-mutant cells 1 Cohort: 12 cycles (daily for 5 days) intervals 4 weeks (*n* = 7) 2 Cohort: 1x week for 24 weeks (*n* = 12) 3 Cohort: 5days + 1x week for 5 weeks (*n* = 3)	Histological Protein biomarker: p53, Ki67 and cyclin D1	HR: 37% p53 (*p* = 0.027).	Stomatitis, diarrhea, fatigue, pain, fever, flu symptoms, dysphagia, vertigo, and infection	30 months
Mulshine et al., (44) RCT	Ketorolac rinse solution 0.1% 10 ml (*n* = 38) Placebo (*n* = 19) 2x day for 33 days	Clinical and histological	CR: Ketorolac 10.8% Placebo 5.2% (*p* = 0.88) HR: Ketorolac (*p* = 0.76) Placebo (*p* = 0.80)	Pain (*n* = 1)	3 months
Scardina et al., (43) CT	Isotretinoin 0,18% gel (*n* = 40) 2x day for 3 months	Clinical	CR: 85%	Transient erythema and xerostomia	120 months
Mallery et al., (45) RCT	Dried black raspberries (DBR) 10% gel (*n* = 21) Placebo (*n* = 19) for 3 months	Clinical, histological Molecular biomarkers: LOH	CR: DBR 76% (*p* = 0.0019) Placebo 10% (*p* = 0.0395) HR: DBR 40.9% (*p* = 0.0488) Placebo 38.9% (*p* = 0.4961) LOH: DBR 45.5% (*p* = 0.002) Placebo 38.9% (*p* = 0.1602)	None	3 months

CS, case series; CT, clinical trial; RCT, randomized clinical trial; CT, clinical trial; CR, clinical response; HR, histological response; LOH, loss of heterozygosity.

Two studies evaluated the effect of topical bleomycin on OL using different concentrations for 2 weeks. Wong et al. (39) observed that only two out of five cases treated with bleomycin 0.5% showed partial clinical response, and there was no histological improvement. When a higher dose of 1% was tested, three out of seven achieved complete clinical and histological regression, indicating a dose-dependent relation (39). In the RCT by Epstein et al. (40), bleomycin 1% was superior to placebo only regarding clinical response, and not for the histological outcome (40).

Two trials evaluated the effect of topical isotretinoin 0.1% applied three times a day for four months (41, 42). One study observed complete clinical regression in 10% of the lesions (41), while the investigation by Tetè et al. (42) observed three complete clinical regressions (42). Both studies detected a statistical increase of apoptotic bodies, but no difference for bcl-2 expression. Scardina et al. (43) observed complete clinical regression in 85% of lesions after treatment with isotretinoin gel 0.18% twice a day for two months. After a follow-up period of 10 years, the study found no cases of MT (43).

Rudin et al. (13) evaluated a mouthwash with ONYX-015, an adenovirus vector targeting p53-mutant cells. Histological resolution was achieved in 37% of patients, correlating with decreased p53 positivity over time. No significant changes regarding cyclin D1 or Ki-67 expression were found, suggesting that these are not predictive of response to ONYX-015 (13). In an RCT conducted by Mulshine et al. (44) the efficacy of chemoprevention of oropharyngeal leukoplakia using a rinse solution containing 0.1% Ketorolac, a cyclooxygenase inhibitor, was evaluated. Ketorolac was administered twice a day for 33 days, but clinical and histological outcomes did not demonstrate efficacy compared to placebo (44). Mallery et al. (45) evaluated a bioadhesive gel containing 10% freeze-dried black raspberry (BRB) for three months. The results showed significant clinical regression of the lesions and reduced histological grades and LOH events without side effects (45). The bioadhesive gel containing BRB appears to be a promising topical chemopreventive strategy.

## 4. Discussion

### 4.1. The challenges of oral leukoplakia chemoprevention

There is very little research exploring the potential of chemoprevention of oral cancer, and many of the studies available present significant methodological issues. An important highlight is that many of these studies are not RCTs. Consequently, there is a risk of biases in both patient selection and outcome assessment. Furthermore, the sample sizes in numerous studies are insufficient, challenging the establishment of conclusive findings (13, 41, 42).

There are no standards regarding outcome assessment in this field. While most studies have assessed clinical and/or histological resolution, it should be noted that these analyses may not necessarily indicate a significant impact on the carcinogenesis process. When performing histological analysis, epithelial dysplasia severity is usually assessed. However, this assessment is subject to significant intra- and inter-subjectivity, which is still considered a major issue among pathologists (46).To improve the consistency and reliability of diagnoses, it is advisable to incorporate a binary classification system like the one suggested by Kujan et al. (47) in addition to the conventional WHO system (47). It can reduce confusion and enhance uniformity in the interpretation of findings. Recent studies have included molecular analysis as an outcome measure (35, 36, 45), leading to more effective results. Thus, the main mutations linked to oral carcinogenesis can be assessed, and the risk of cancer development can be better predicted. With the next-generation sequencing approach becoming more readily available, its use will unveil the effects of chemopreventive agents at the molecular level. Consequently, it will allow accurate monitoring of alterations in primary oncogenic mutations or epigenetic modifications (48–50).

Nevertheless, regarding leukoplakia chemoprevention, the ideal outcome is cancer-free survival, which requires a long follow-up and a large sample size. In this sense, multicenter studies are desirable as they can gather a higher number of patients. Moreover, multicenter studies are advantageous as they increase sample heterogeneity and enable the analysis of the efficacy of chemopreventive agents in different populations (36).

Currently, there are limited chemopreventive options available for OL and those that have been evaluated frequently result in significant side effects. Systemic agents offer the advantage of a more widespread effect, which is especially relevant considering the existence of a cancerization field in the oral cavity. This means that altered cells can be found beyond clinically visible lesions (51). Nevertheless, it is essential to note that systemic drugs carry a higher risk of toxicity and side effects, including gastrointestinal symptoms, liver toxicity, increased risk of bleeding, among others. As such, it is crucial to carefully monitor individuals receiving chemopreventive agents for any adverse effects and adjust the dosage or discontinue the agent if necessary. These undesirable effects may be more relevant for patients with comorbidities or who are taking other medications regularly. Therefore, the cost-benefit ratio must be individually assessed.

Topical agents, on the other hand, present a lower risk of significant side effects since they act locally. However, delivering a substance to the oral mucosa has additional challenges. Firstly, some lesions may be located in subsites that are difficult to reach. Secondly, due to the salivary flow, the residence time of drugs in conventional formulations is reduced, potentially decreasing their effectiveness. Therefore, the development of mucoadhesive delivery systems is necessary to achieve better results.

When considering a potential chemoprevention agent, it is essential to also consider their long-term safety since prevention may require longer periods of treatment (31, 33, 35). This is particularly important as prolonged exposure may lead to unexpected adverse effects or toxicity. Moreover, it is important to note that resistance to chemopreventive agents can develop over time, making them less effective.

Considering the criteria of response to treatment, follow-up time, and severity of side effects from studies included in this review, it can be suggested that among systemic chemopreventives, lycopene (28) and green tea extract (GTE) (30) appear to be promising. Both 8 mg and 4 mg lycopene capsules caused histological response, without side effects, although the follow-up time was short, only 2 months. GTE also showed a histological improvement, the side effects reported were mild and a long follow-up period of 27.5 months was reported. Among topical chemopreventives, the application of 10% dried black raspberries (BRB) bioadhesive gel (45) seems promising, showing a histological improvement of lesions and no adverse effects, although the follow-up time was only 3 months. However, it is important to note that these chemopreventive agents were evaluated in a limited number of studies, and additional studies with larger samples and longer follow-up times are needed to confirm these findings.

Even after addressing all the issues raised so far, there are still several challenges associated with implementing chemoprevention for OL on a large scale. Firstly, there is poor awareness about the benefits of chemoprevention and the risk factors for OL, which can result in low participation rates. Early detection of OL remains a challenge, hindering effective implementation of chemoprevention strategies. Additionally, chemoprevention can be expensive and may not be covered by insurance, making it unaffordable to many patients. Nevertheless, healthcare systems and their administrators should consider that the treatment for oral cancer would be much more expensive than its chemoprevention, thus justifying the implementation of these strategies. This highlights the importance of raising awareness of the benefits of chemoprevention and making it more accessible to those who need it, ultimately leading to a decrease in the incidence and burden of oral cancer.

### 4.2. What can we improve in the search for an ideal chemopreventive for oral leukoplakia?

To improve the search for an ideal chemopreventive for treating OL, several strategies can be employed. Firstly, there is a need for a better understanding of the molecular mechanisms involved in oral carcinogenesis to identify the most potential targets for chemoprevention. Secondly, it is crucial to focus on developing safe and effective chemopreventive agents with minimal side effects. Thirdly, there is a need for more standardized RCTs to evaluate the efficacy and safety of potential chemopreventive agents. The future studies on this subject should focus on the identification and selection of high-risk individuals, based on their profile, the subsite of the lesion, use of carcinogenic agents, and especially molecular markers such as LOH and mutations in the main pathways of oral carcinogenesis (35, 36, 52). Also, there should be efforts to increase public awareness about the benefits of chemoprevention and risk factors for oral cancer to increase participation rates. This can be achieved through education and awareness campaigns, targeted screening programs, and community outreach programs. A greater collaboration among researchers, clinicians, policymakers, and other stakeholders is also necessary to implement effective chemoprevention programs at the population level. This may involve various measures such as creating national guidelines for oral cancer screening and prevention, integrating chemoprevention into existing cancer prevention and control programs, and promoting public-private partnerships to facilitate research and development of new chemopreventive agents.

## 5. Conclusion

In summary, OL chemoprevention presents a promising strategy to reduce the incidence of oral cancer in high-risk individuals. Investigating chemopreventive agents that target molecular pathways implicated in cancer development and progression in clinical trials is crucial. Careful patient selection and monitoring for potential side effects are necessary for the safe and efficient implementation of chemopreventive agents. Furthermore, identifying novel chemopreventive agents and biomarkers for predicting treatment response should be a priority of future research.
